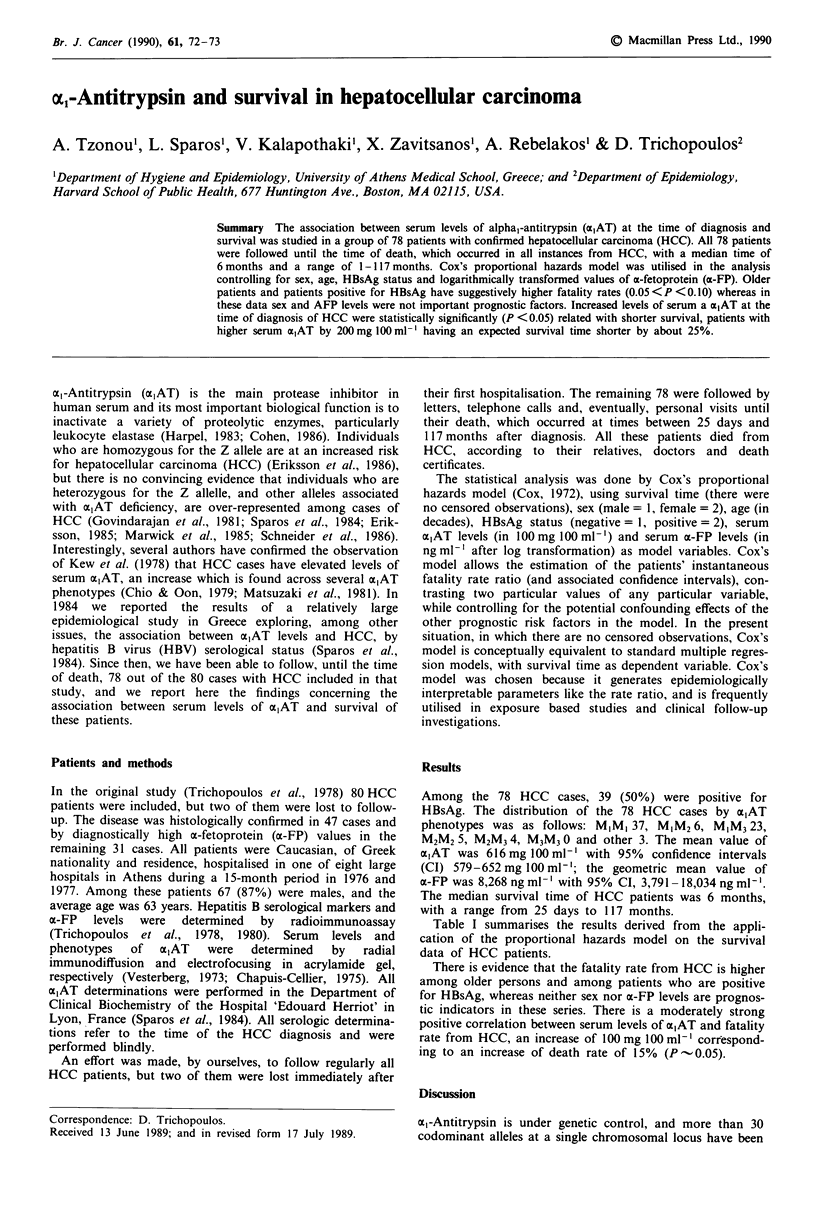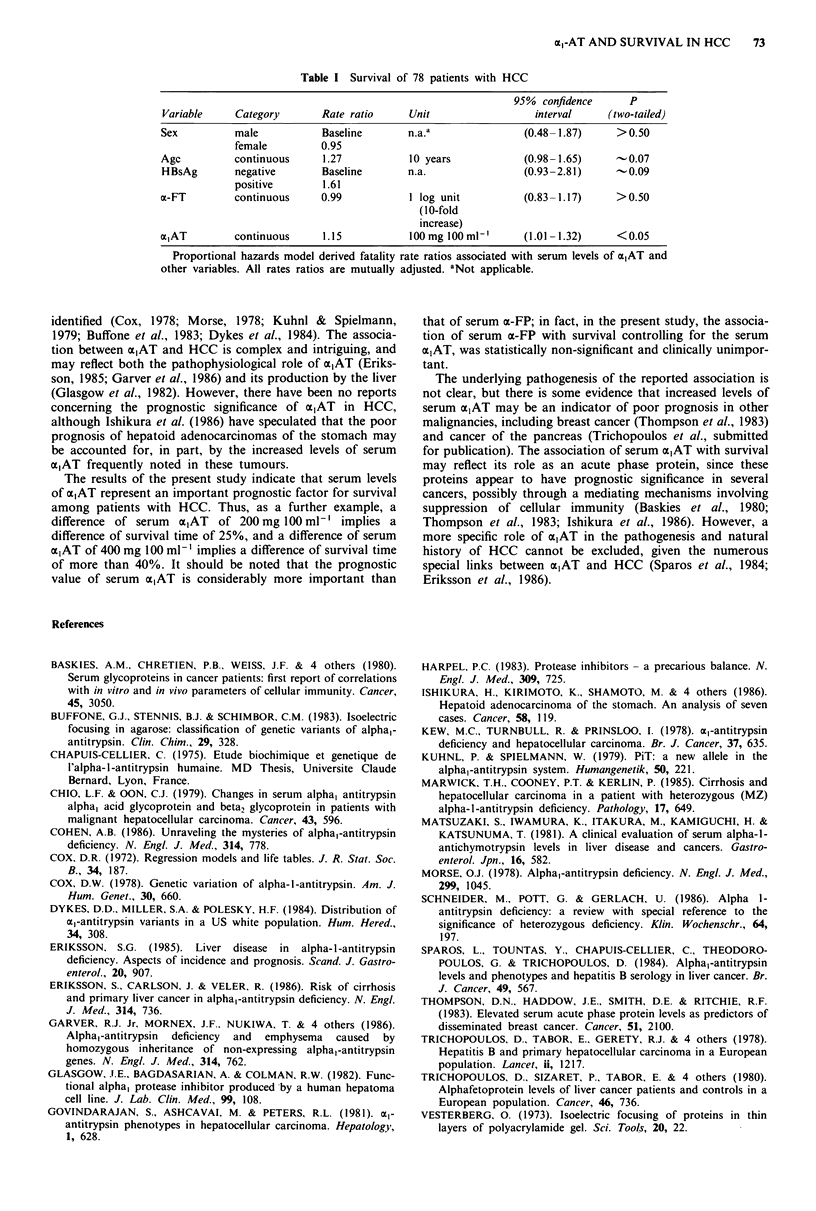# Alpha 1-antitrypsin and survival in hepatocellular carcinoma.

**DOI:** 10.1038/bjc.1990.16

**Published:** 1990-01

**Authors:** A. Tzonou, L. Sparos, V. Kalapothaki, X. Zavitsanos, A. Rebelakos, D. Trichopoulos

**Affiliations:** Department of Hygiene and Epidemiology, University of Athens Medical School, Greece.

## Abstract

The association between serum levels of alpha 1-antitrypsin (alpha 1 AT) at the time of diagnosis and survival was studied in a group of 78 patients with confirmed hepatocellular carcinoma (HCC). All 78 patients were followed until the time of death, which occurred in all instances from HCC, with a median time of 6 months and a range of 1-117 months. Cox's proportional hazards model was utilised in the analysis controlling for sex, age, HBsAg status and logarithmically transformed values of alpha-fetoprotein (alpha-FP). Older patients and patients positive for HBsAg have suggestively higher fatality rates (0.05 less than P less than 0.10) whereas in these data sex and AFP levels were not important prognostic factors. Increased levels of serum at alpha 1AT at the time of diagnosis of HCC were statistically significantly (P less than 0.05) related with shorter survival, patients with higher serum alpha 1AT by 200 mg 100 ml-1 having an expected survival time shorter by about 25%.


 © Macmillan Press Ltd., 1990

### $\alpha_1$-Antitrypsin and survival in hepatocellular carcinoma

A. Tzonou[1], L. Sparos[1], V. Kalapothaki[1], X. Zavitsanos[1], A. Rebelakos[1] & D. Trichopoulos[2]

[1]*Department of Hygiene and Epidemiology, University of Athens Medical School, Greece; and* [2]*Department of Epidemiology, Harvard School of Public Health, 677 Huntington Ave., Boston, MA 02115, USA.*

**Summary** The association between serum levels of alpha$_1$-antitrypsin ($\alpha_1$AT) at the time of diagnosis and survival was studied in a group of 78 patients with confirmed hepatocellular carcinoma (HCC). All 78 patients were followed until the time of death, which occurred in all instances from HCC, with a median time of 6 months and a range of 1–117 months. Cox's proportional hazards model was utilised in the analysis controlling for sex, age, HBsAg status and logarithmically transformed values of $\alpha$-fetoprotein ($\alpha$-FP). Older patients and patients positive for HBsAg have suggestively higher fatality rates ($0.05 < P < 0.10$) whereas in these data sex and AFP levels were not important prognostic factors. Increased levels of serum a $\alpha_1$AT at the time of diagnosis of HCC were statistically significantly ($P < 0.05$) related with shorter survival, patients with higher serum $\alpha_1$AT by 200 mg 100 ml$^{-1}$ having an expected survival time shorter by about 25%.

$\alpha_1$-Antitrypsin ($\alpha_1$AT) is the main protease inhibitor in human serum and its most important biological function is to inactivate a variety of proteolytic enzymes, particularly leukocyte elastase (Harpel, 1983; Cohen, 1986). Individuals who are homozygous for the Z allele are at an increased risk for hepatocellular carcinoma (HCC) (Eriksson *et al.*, 1986), but there is no convincing evidence that individuals who are heterozygous for the Z allelle, and other alleles associated with $\alpha_1$AT deficiency, are over-represented among cases of HCC (Govindarajan *et al.*, 1981; Sparos *et al.*, 1984; Eriksson, 1985; Marwick *et al.*, 1985; Schneider *et al.*, 1986). Interestingly, several authors have confirmed the observation of Kew *et al.* (1978) that HCC cases have elevated levels of serum $\alpha_1$AT, an increase which is found across several $\alpha_1$AT phenotypes (Chio & Oon, 1979; Matsuzaki *et al.*, 1981). In 1984 we reported the results of a relatively large epidemiological study in Greece exploring, among other issues, the association between $\alpha_1$AT levels and HCC, by hepatitis B virus (HBV) serological status (Sparos *et al.*, 1984). Since then, we have been able to follow, until the time of death, 78 out of the 80 cases with HCC included in that study, and we report here the findings concerning the association between serum levels of $\alpha_1$AT and survival of these patients.

### Patients and methods

In the original study (Trichopoulos *et al.*, 1978) 80 HCC patients were included, but two of them were lost to follow-up. The disease was histologically confirmed in 47 cases and by diagnostically high $\alpha$-fetoprotein ($\alpha$-FP) values in the remaining 31 cases. All patients were Caucasian, of Greek nationality and residence, hospitalised in one of eight large hospitals in Athens during a 15-month period in 1976 and 1977. Among these patients 67 (87%) were males, and the average age was 63 years. Hepatitis B serological markers and $\alpha$-FP levels were determined by radioimmunoassay (Trichopoulos *et al.*, 1978, 1980). Serum levels and phenotypes of $\alpha_1$AT were determined by radial immunodiffusion and electrofocusing in acrylamide gel, respectively (Vesterberg, 1973; Chapuis-Cellier, 1975). All $\alpha_1$AT determinations were performed in the Department of Clinical Biochemistry of the Hospital 'Edouard Herriot' in Lyon, France (Sparos *et al.*, 1984). All serologic determinations refer to the time of the HCC diagnosis and were performed blindly.

An effort was made, by ourselves, to follow regularly all HCC patients, but two of them were lost immediately after their first hospitalisation. The remaining 78 were followed by letters, telephone calls and, eventually, personal visits until their death, which occurred at times between 25 days and 117 months after diagnosis. All these patients died from HCC, according to their relatives, doctors and death certificates.

The statistical analysis was done by Cox's proportional hazards model (Cox, 1972), using survival time (there were no censored observations), sex (male = 1, female = 2), age (in decades), HBsAg status (negative = 1, positive = 2), serum $\alpha_1$AT levels (in 100 mg 100 ml$^{-1}$) and serum $\alpha$-FP levels (in ng ml$^{-1}$ after log transformation) as model variables. Cox's model allows the estimation of the patients' instantaneous fatality rate ratio (and associated confidence intervals), contrasting two particular values of any particular variable, while controlling for the potential confounding effects of the other prognostic risk factors in the model. In the present situation, in which there are no censored observations, Cox's model is conceptually equivalent to standard multiple regression models, with survival time as dependent variable. Cox's model was chosen because it generates epidemiologically interpretable parameters like the rate ratio, and is frequently utilised in exposure based studies and clinical follow-up investigations.

### Results

Among the 78 HCC cases, 39 (50%) were positive for HBsAg. The distribution of the 78 HCC cases by $\alpha_1$AT phenotypes was as follows: $M_1M_1$ 37, $M_1M_2$ 6, $M_1M_3$ 23, $M_2M_2$ 5, $M_2M_3$ 4, $M_3M_3$ 0 and other 3. The mean value of $\alpha_1$AT was 616 mg 100 ml$^{-1}$ with 95% confidence intervals (CI) 579–652 mg 100 ml$^{-1}$; the geometric mean value of $\alpha$-FP was 8,268 ng ml$^{-1}$ with 95% CI, 3,791–18,034 ng ml$^{-1}$. The median survival time of HCC patients was 6 months, with a range from 25 days to 117 months.

Table I summarises the results derived from the application of the proportional hazards model on the survival data of HCC patients.

There is evidence that the fatality rate from HCC is higher among older persons and among patients who are positive for HBsAg, whereas neither sex nor $\alpha$-FP levels are prognostic indicators in these series. There is a moderately strong positive correlation between serum levels of $\alpha_1$AT and fatality rate from HCC, an increase of 100 mg 100 ml$^{-1}$ corresponding to an increase of death rate of 15% ($P \sim 0.05$).

### Discussion

$\alpha_1$-Antitrypsin is under genetic control, and more than 30 codominant alleles at a single chromosomal locus have been

Correspondence: D. Trichopoulos.
Received 13 June 1989; and in revised form 17 July 1989.

**Table I** Survival of 78 patients with HCC

| *Variable* | *Category* | *Rate ratio* | *Unit* | *95% confidence interval* | *P (two-tailed)* |
|---|---|---|---|---|---|
| Sex | male | Baseline | n.a.[a] | (0.48–1.87) | >0.50 |
| | female | 0.95 | | | |
| Age | continuous | 1.27 | 10 years | (0.98–1.65) | ~0.07 |
| HBsAg | negative | Baseline | n.a. | (0.93–2.81) | ~0.09 |
| | positive | 1.61 | | | |
| α-FT | continuous | 0.99 | 1 log unit (10-fold increase) | (0.83–1.17) | >0.50 |
| $\alpha_1$AT | continuous | 1.15 | 100 mg 100 ml$^{-1}$ | (1.01–1.32) | <0.05 |

Proportional hazards model derived fatality rate ratios associated with serum levels of $\alpha_1$AT and other variables. All rates ratios are mutually adjusted. [a]Not applicable.

identified (Cox, 1978; Morse, 1978; Kuhnl & Spielmann, 1979; Buffone *et al.*, 1983; Dykes *et al.*, 1984). The association between $\alpha_1$AT and HCC is complex and intriguing, and may reflect both the pathophysiological role of $\alpha_1$AT (Eriksson, 1985; Garver *et al.*, 1986) and its production by the liver (Glasgow *et al.*, 1982). However, there have been no reports concerning the prognostic significance of $\alpha_1$AT in HCC, although Ishikura *et al.* (1986) have speculated that the poor prognosis of hepatoid adenocarcinomas of the stomach may be accounted for, in part, by the increased levels of serum $\alpha_1$AT frequently noted in these tumours.

The results of the present study indicate that serum levels of $\alpha_1$AT represent an important prognostic factor for survival among patients with HCC. Thus, as a further example, a difference of serum $\alpha_1$AT of 200 mg 100 ml$^{-1}$ implies a difference of survival time of 25%, and a difference of serum $\alpha_1$AT of 400 mg 100 ml$^{-1}$ implies a difference of survival time of more than 40%. It should be noted that the prognostic value of serum $\alpha_1$AT is considerably more important than that of serum α-FP; in fact, in the present study, the association of serum α-FP with survival controlling for the serum $\alpha_1$AT, was statistically non-significant and clinically unimportant.

The underlying pathogenesis of the reported association is not clear, but there is some evidence that increased levels of serum $\alpha_1$AT may be an indicator of poor prognosis in other malignancies, including breast cancer (Thompson *et al.*, 1983) and cancer of the pancreas (Trichopoulos *et al.*, submitted for publication). The association of serum $\alpha_1$AT with survival may reflect its role as an acute phase protein, since these proteins appear to have prognostic significance in several cancers, possibly through a mediating mechanisms involving suppression of cellular immunity (Baskies *et al.*, 1980; Thompson *et al.*, 1983; Ishikura *et al.*, 1986). However, a more specific role of $\alpha_1$AT in the pathogenesis and natural history of HCC cannot be excluded, given the numerous special links between $\alpha_1$AT and HCC (Sparos *et al.*, 1984; Eriksson *et al.*, 1986).

### References

BASKIES, A.M., CHRETIEN, P.B., WEISS, J.F. & 4 others (1980). Serum glycoproteins in cancer patients: first report of correlations with *in vitro* and *in vivo* parameters of cellular immunity. *Cancer*, **45**, 3050.

BUFFONE, G.J., STENNIS, B.J. & SCHIMBOR, C.M. (1983). Isoelectric focusing in agarose: classification of genetic variants of alpha$_1$-antitrypsin. *Clin. Chim.*, **29**, 328.

CHAPUIS-CELLIER, C. (1975). Etude biochimique et genetique de l'alpha-1-antitrypsin humaine. MD Thesis, Universite Claude Bernard, Lyon, France.

CHIO, L.F. & OON, C.J. (1979). Changes in serum alpha$_1$ antitrypsin alpha$_1$ acid glycoprotein and beta$_2$ glycoprotein in patients with malignant hepatocellular carcinoma. *Cancer*, **43**, 596.

COHEN, A.B. (1986). Unraveling the mysteries of alpha$_1$-antitrypsin deficiency. *N. Engl. J. Med.*, **314**, 778.

COX, D.R. (1972). Regression models and life tables. *J. R. Stat. Soc. B.*, **34**, 187.

COX, D.W. (1978). Genetic variation of alpha-1-antitrypsin. *Am. J. Hum. Genet.*, **30**, 660.

DYKES, D.D., MILLER, S.A. & POLESKY, H.F. (1984). Distribution of $\alpha_1$-antitrypsin variants in a US white population. *Hum. Hered.*, **34**, 308.

ERIKSSON, S.G. (1985). Liver disease in alpha-1-antitrypsin deficiency. Aspects of incidence and prognosis. *Scand. J. Gastroenterol.*, **20**, 907.

ERIKSSON, S., CARLSON, J. & VELER, R. (1986). Risk of cirrhosis and primary liver cancer in alpha$_1$-antitrypsin deficiency. *N. Engl. J. Med.*, **314**, 736.

GARVER, R.J. Jr, MORNEX, J.F., NUKIWA, T. & 4 others (1986). Alpha$_1$-antitrypsin deficiency and emphysema caused by homozygous inheritance of non-expressing alpha$_1$-antitrypsin genes. *N. Engl. J. Med.*, **314**, 762.

GLASGOW, J.E., BAGDASARIAN, A. & COLMAN, R.W. (1982). Functional alpha$_1$ protease inhibitor produced by a human hepatoma cell line. *J. Lab. Clin. Med.*, **99**, 108.

GOVINDARAJAN, S., ASHCAVAI, M. & PETERS, R.L. (1981). $\alpha_1$-antitrypsin phenotypes in hepatocellular carcinoma. *Hepatology*, **1**, 628.

HARPEL, P.C. (1983). Protease inhibitors – a precarious balance. *N. Engl. J. Med.*, **309**, 725.

ISHIKURA, H., KIRIMOTO, K., SHAMOTO, M. & 4 others (1986). Hepatoid adenocarcinoma of the stomach. An analysis of seven cases. *Cancer*, **58**, 119.

KEW, M.C., TURNBULL, R. & PRINSLOO, I. (1978). $\alpha_1$-antitrypsin deficiency and hepatocellular carcinoma. *Br. J. Cancer*, **37**, 635.

KUHNL, P. & SPIELMANN, W. (1979). PiT: a new allele in the alpha$_1$-antitrypsin system. *Humangenetik*, **50**, 221.

MARWICK, T.H., COONEY, P.T. & KERLIN, P. (1985). Cirrhosis and hepatocellular carcinoma in a patient with heterozygous (MZ) alpha-1-antitrypsin deficiency. *Pathology*, **17**, 649.

MATSUZAKI, S., IWAMURA, K., ITAKURA, M., KAMIGUCHI, H. & KATSUNUMA, T. (1981). A clinical evaluation of serum alpha-1-antichymotrypsin levels in liver disease and cancers. *Gastroenterol. Jpn.*, **16**, 582.

MORSE, O.J. (1978). Alpha$_1$-antitrypsin deficiency. *N. Engl. J. Med.*, **299**, 1045.

SCHNEIDER, M., POTT, G. & GERLACH, U. (1986). Alpha 1-antitrypsin deficiency: a review with special reference to the significance of heterozygous deficiency. *Klin. Wochenschr.*, **64**, 197.

SPAROS, L., TOUNTAS, Y., CHAPUIS-CELLIER, C., THEODOROPOULOS, G. & TRICHOPOULOS, D. (1984). Alpha$_1$-antitrypsin levels and phenotypes and hepatitis B serology in liver cancer. *Br. J. Cancer*, **49**, 567.

THOMPSON, D.N., HADDOW, J.E., SMITH, D.E. & RITCHIE, R.F. (1983). Elevated serum acute phase protein levels as predictors of disseminated breast cancer. *Cancer*, **51**, 2100.

TRICHOPOULOS, D., TABOR, E., GERETY, R.J. & 4 others (1978). Hepatitis B and primary hepatocellular carcinoma in a European population. *Lancet*, **ii**, 1217.

TRICHOPOULOS, D., SIZARET, P., TABOR, E. & 4 others (1980). Alphafetoprotein levels of liver cancer patients and controls in a European population. *Cancer*, **46**, 736.

VESTERBERG, O. (1973). Isoelectric focusing of proteins in thin layers of polyacrylamide gel. *Sci. Tools*, **20**, 22.